# Recent Advances in Pyridine Scaffold: Focus on Chemistry, Synthesis, and Antibacterial Activities

**DOI:** 10.1155/2023/9967591

**Published:** 2023-05-18

**Authors:** Md. Badrul Islam, Md. Inshaful Islam, Nikhil Nath, Talha Bin Emran, Md. Rezaur Rahman, Rohit Sharma, Mohammed Mahbubul Matin

**Affiliations:** ^1^Bioorganic and Medicinal Chemistry Laboratory, Department of Chemistry, Faculty of Science, University of Chittagong, Hathazari, Chittagong 4331, Bangladesh; ^2^Department of Pharmacy, International Islamic University Chittagong, Chittagong 4318, Bangladesh; ^3^Department of Pharmacy, BGC Trust University Bangladesh, Chittagong 4381, Bangladesh; ^4^Department of Pharmacy, Faculty of Allied Health Sciences, Daffodil International University, Dhaka 1207, Bangladesh; ^5^Department of Chemical Engineering and Energy Sustainability, Faculty of Engineering, Universiti Malaysia Sarawak, Jalan Datuk Mohammad Musa, Kota Samarahan 94300, Malaysia; ^6^Department of Rasa Shastra and Bhaishajya Kalpana, Faculty of Ayurveda, Institute of Medical Sciences, Banaras Hindu University, Varanasi, 221005 Uttar Pradesh, India

## Abstract

Multidrug-resistant (MDR) pathogens have created a fatal problem for human health and antimicrobial treatment. Among the currently available antibiotics, many are inactive against MDR pathogens. In this context, heterocyclic compounds/drugs play a vital role. Thus, it is very much essential to explore new research to combat the issue. Of the available nitrogen-bearing heterocyclic compounds/drugs, pyridine derivatives are of special interest due to their solubility. Encouragingly, some of the newly synthesized pyridine compounds/drugs are found to inhibit multidrug-resistant *S. aureus* (MRSA). Pyridine scaffold bearing poor basicity generally improves water solubility in pharmaceutically potential molecules and has led to the discovery of numerous broad-spectrum therapeutic agents. Keeping these in mind, we have reviewed the chemistry, recent synthetic techniques, and bacterial preventative activity of pyridine derivatives since 2015. This will facilitate the development of pyridine-based novel antibiotic/drug design in the near future as a versatile scaffold with limited side effects for the next-generation therapeutics.

## 1. Introduction

Most of the nitrogen-bearing heterocyclic compounds are biologically potential [1–3] and play a vital role in progressive drug design and discovery [4]. Among them, the six-membered heteroaromatic pyridine nucleus is ubiquitous and found in natural sources such as alkaloids (nicotine), vitamins (niacin and pyridoxine), and coenzymes [5]. Although pyridine is a very common solvent in organic laboratories, its derivatives have diverse applications in functional nanomaterials, as important ligands for organometallic compounds, and in asymmetric catalysis [6, 7]. In organic chemistry, pyridine and its derivatives play vital roles [8] and are the most extensively applied scaffolds for drug design and synthesis. In fact, pyridine scaffold-containing compounds have received significant interest in multiple research fields. This is mainly related to their (*i*) unique heteroaromatic functional role in organic chemistry, (*ii*) easy conversion into different functional derivatives, (*iii*) profound effect on pharmacological activity, and (*iv*) application as pharmacophores in medicinal chemistry [9]. These properties led to the discovery of many broad-spectrum therapeutic agents [10] and agrochemical products [11].

**Figure 1:**
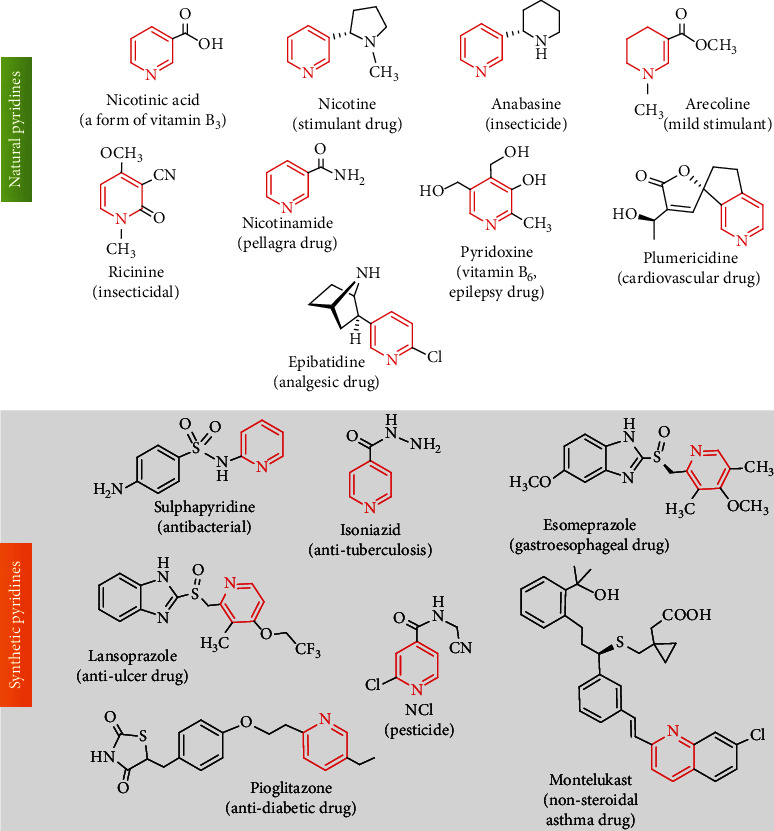
Pyridine scaffold-bearing drugs in therapeutic applications.

Privileged with the pyridine scaffold, many drugs have been synthesized/discovered and several of them are on the market as therapeutic drugs. Hundreds of compounds with pyridine scaffolds are listed as drugs (https://go.drugbank.com/categories/DBCAT000227) including FDA [5]. For example, the attachment of this auspice nucleus to sulfanilamide produced an antibacterial antibiotic named sulfapyridine (Figure 1). The most important pyridine-based therapeutic drugs are isoniazid (brand name: Nydrazid; an antibiotic for tuberculosis), esomeprazole (brand name: Nexium), lansoprazole (brand name: Takepron), montelukast (brand name: Singulair), pioglitazone (brand name: Actos), etc. (Figure 1). In addition, low molecular weight antibacterial drugs like ozenoxacin and ethionamide are notable [12, 13].

Many naturally occurring compounds are reported to possess a pyridine nucleus. These are considered alkaloids, and notable examples are plumericidine (from *Plumeria rubra* L., used for cardiovascular treatment [14]), dictamnine-7-*β*-D-mannopyranoside (from *Solidago canadensis* [15]), epibatidine (from *Epipedobates tricolor* skin, an analgesic agent [16]), and pyrinadine A (a bis-pyridine alkaloid from *Cribochalina* sp., anticarcinogenic and antiparasitic [17]).

Insertion of a simple COOH group at the C-3 position of pyridine (as in nicotinic acid or niacin, the oldest drug for dyslipidemia) enhances pyridine's bioactivity, and niacin was reported as a precursor for many other significant bioactive molecules (such as NAD^+^ and NADP^+^ [18]). In general, the incorporation/fusion of other rings, especially heterocyclic ring(s) with the pyridine nucleus, enhances its bioactivity and intensifies its antimicrobial properties [4, 19]. Additional attachment of several functional groups (amino, hydroxy, methoxy, sulfamide, hydrazide, etc.) enhances the compound's bioactivity further [20].

Thus, pyridine scaffold compounds and materials are valued for their biological, medicinal, optical, chemical, and physical properties among nitrogen-based heterocycles. This review is aimed at focusing on chemistry and the reported synthetic methods of pyridine scaffolds since 2015, emphasizing the antibacterial potential.

## 2. Chemistry of Pyridine Compounds

**Figure 2:**
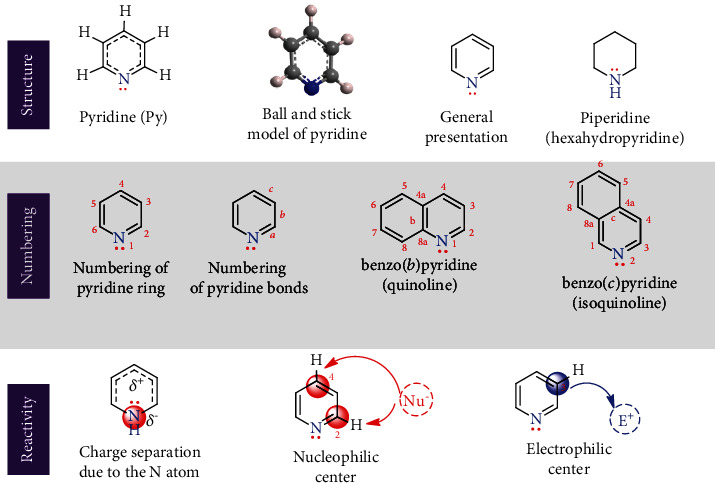
Structure, numbering, and active sites of pyridine.

Mononitrogen containing a six-membered heteroaromatic compound structurally similar to benzene with the molecular formula C_5_H_5_N is named pyridine (Py, Figure 2). It is also known as monoazabenzene, azaarene, azine, etc. and is the parent compound of the class pyridines. This simplest and most common compound is water-miscible, flammable, and colorless to yellow liquid (bp 115.5°C and mp -41.6°C). Due to water miscibility, it is used to dissolve other substances. However, pyridine bears an unpleasant/foul smell and some hazardous properties.

Cyclic pyridine is planar with a *sp*^2^ hybridized *N* atom and five *C* atoms and has a delocalized pi-molecular orbital that fulfills the Hückel criteria ((4*n* + 2)*π* electrons) and thus confirms its aromaticity. Structurally, pyridine is isoelectronic with benzene and exhibits unusual isotopic polymorphism properties [21]. However, its physical and chemical properties are quite different from benzene. Unlike neutral benzene, pyridine is a weak base and behaves as a tertiary amine in many respects [22]. The presence of the nonconjugated lone pair electrons on the *sp*^2^ hybridized nitrogen atom governs pyridine's basicity. In fact, its basicity is greater than *N*,*N*-dimethylaniline and weaker than aliphatic 3° amines. It can form hydrogen bonding and utilizes lone pair n(N) or *π*-electrons. This property governs its interaction with other substances, enzymes, etc. and is a viable factor for its regioselective and catalytic C–H functionalization [23–25].

Due to pyridine's basic nature, it can form stable salts when treated with stronger acids or alkyl halides (the Menshutkin reaction). In addition, it can be used to neutralize some acids produced in chemical reactions. Like other aromatic compounds, pyridine is more prone to substitution reactions. However, it prefers nucleophilic substitution (at the C-2 and C-4 positions) to electrophilic substitution (at the C-3 position under drastic reaction conditions) because of the –I effect of the ring nitrogen (Figure 2). This –I effect enables the nitrogen atom to become electron-rich and the aromatic ring to become electron-deficient, i.e., charge separation in the ring.

## 3. Synthetic Approach for Biologically Significant Pyridine Compounds

Because of its properties, including basicity, water solubility, stability, the capacity to establish hydrogen bonds, and its tiny molecular size, pyridine moieties are frequently utilized in pharmaceuticals [26–28]. Hence, several innovative synthetic methods have been developed in the last couple of years for both (*a*) substituted pyridines and (*b*) ring-fused pyridines. The significant strategies are discussed here.

### 3.1. Substituted Pyridine Analogs

**Scheme 1:**
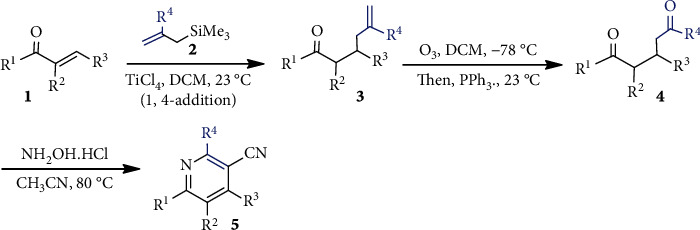
Synthetic route to substituted pyridine ring.

Focusing on pharmaceutical importance, Hilf et al. [29] reported a three-step route leading to highly substituted pyridines. Initially, 1,5-dicarbonyls **4** are prepared from enones **3** (obtained from **1** and **2**) via a two-step Hosomi-Sakurai allylation/oxidative cleavage sequence (Scheme 1). Diketo compound **4** upon cyclization with hydroxylamine hydrochloride furnishes substituted pyridines **5**.

Several more synthetic methods are reported for the diversely substituted pyridine preparation. Methods related to biological/pharmaceutical potential are presented below.

#### 3.1.1. Catalyst-Mediated Synthesis

**Scheme 2:**
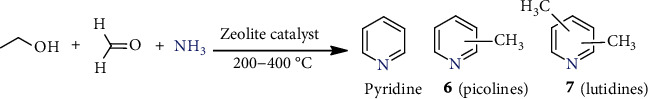
Zeolite catalyzed pyridine synthesis.

Grigor'eva et al. [30] reported zeolite catalysts (H-Beta, H-ZSM-5, and H-ZSM-12) in the three-component condensation leading to pyridine, picolines **6** (2-, 3-, or 4-methylpyridine), and lutidines **7** (dimethylpyridine). The condensation reaction among ethanol, formaldehyde, and ammonia with a zeolite catalyst is shown in Scheme 2. The best efficacy was obtained for H-Beta zeolite. H-Beta and H-ZSM-12 zeolites formed pyridines and picolines, whereas H-ZSM-12 catalyzed the formation of pyridines **6** and **7**.

**Scheme 3:**
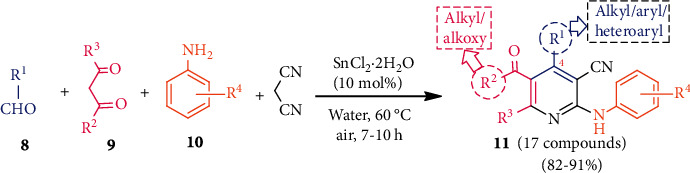
Sn(IV)-catalyzed preparation of substituted pyridines via a MCR reaction.

In the same year, SnCl_2_•2H_2_O was used for the first time for the construction of the pyridine skeleton via a multicomponent reaction in water [31]. Thus, heating a mixture of 4 components such as aldehydes **8**, *β*-keto esters (or 1,3-diketones, **9**), anilines **10**, and malononitrile with Sn(IV) catalyst afforded polysubstituted pyridines **11** in good yields (Scheme 3). This simple method can be utilized for medicinally important substituted pyridines.

**Scheme 4:**
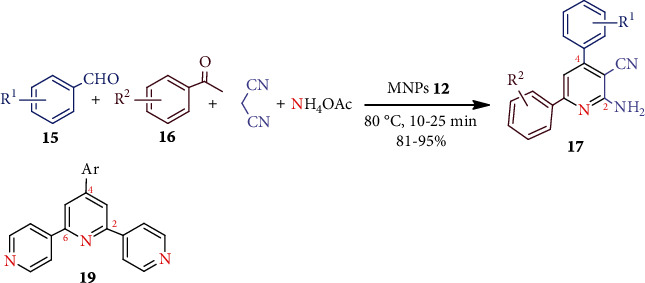
MNPs **11** catalyzed synthesis of 2-amino-4,6-diphenylnicotinonitriles **17**.

Magnetic nanocatalysts, i.e., magnetic nanoparticles (MNPs), have also been applied in multicomponent reactions (MCRs) for pyridine synthesis. This is mainly due to their high surface-to-volume ratios and magnetically recoverable properties. Some common catalysts employed for the pyridine synthesis are Fe_3_O_4_@SiO_2_-pr-NH_2_, Fe_3_O_4_@SiO_2_-morpholine MNPs (**12**), Fe_3_O_4_–Si–(CH_2_)_3_–N]CH–Ph–OMe MNPs (**13**), CoFe_2_O_4_@Silica MNPs (**14**), poly *N*,*N*-dimethylaniline-formaldehyde supported on silica-coated Fe_3_O_4_ MNPs (PDMAF-MNPs), Fe_3_O_4_@CoII (macrocyclic Schiff base ligand), Fe_3_O_4_@SiO_2_@Pr-SO_3_H, etc. [32]. For example, MNPs **12** catalyzed MCR among benzaldehydes (**15**), acetophenone derivatives (**16**), malononitrile, and ammonium acetate in the absence of solvent to furnish 2-amino-4,6-diphenylnicotinonitriles (**17**) (Scheme 4) [33].

Similarly, MNPs **13** [34] or CoFe_2_O_4_@SiO_2_ MNPs (**14**) [35] catalysts were successfully employed for the synthesis of **17 ***via* MCRs. In each case, **17** was obtained with good to high yields in a short reaction time. Encouragingly, ionic MNPs such as Fe_3_O_4_@O_2_PO_2_(CH_2_)_2_NH_3_^+^ CF_3_CO_2_^−^ (**18**) were used for easy access to terpyridines **19** (an important precursor for several antimicrobial agents [36]) using MCRs [37].

**Scheme 5:**
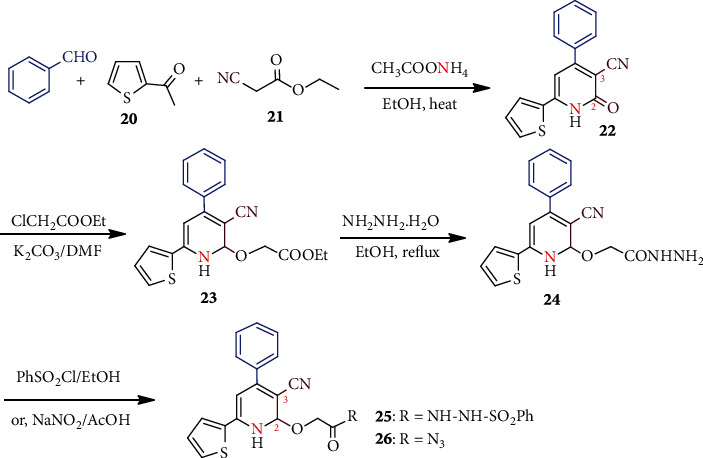
Synthesis of polysubstituted antibacterial 2,3-dihydro-pyridine compounds **25** and **26**.

Metal-free CH_3_COONH_4_-catalyzed polysubstituted pyridine ring formation was reported for further exploitation into several antimicrobial agents [38]. As shown in Scheme 5, the initially substituted skeleton **22** was constructed from the condensation of benzaldehyde, 2-acetylthiophene (**20**), and ethyl cyanoacetate (**21**). The reaction of **22** with ethyl chloroacetate furnished ethyl ester **23**, which on treatment with hydrazine, gave hydrazone **24**. Compound **24** was prepared by separate treatment with several reagents to provide different substituted 2,3-dihydro-2-oxo-pyridine products (only compounds **25** and **26** are shown here). Notably, compounds **25** (59.54%) and **26** (55.84%) exhibited the highest inhibitory property against Gram-positive *S. aureus*.

**Scheme 6:**
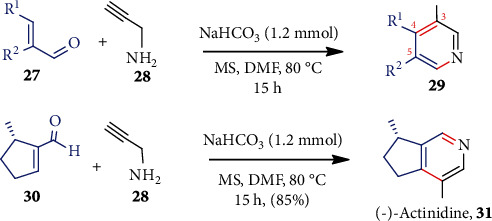
NaHCO_3_ catalyzed substituted pyridines from various unsaturated aldehydes.

Uredi et al. [39] showed a simple, metal-free, and mild strategy for the construction of multisubstituted pyridines with excellent yields (Scheme 6). Thus, condensation between *α*,*β*-unsaturated aldehydes **27** and propargylamine **28** catalyzed by a very cheap NaHCO_3_ furnished **29** and byproduct water only. The condensation proceeds with imine formation and concomitant cyclization via an allenyl intermediate. This protocol can be used for a wider range of *α*,*β*-unsaturated aldehydes. For example, the use of cyclic enal **30** furnished the natural alkaloid (−)-actinidine (**31**).

#### 3.1.2. Microwave- (MW-) Assisted Synthesis

**Scheme 7:**
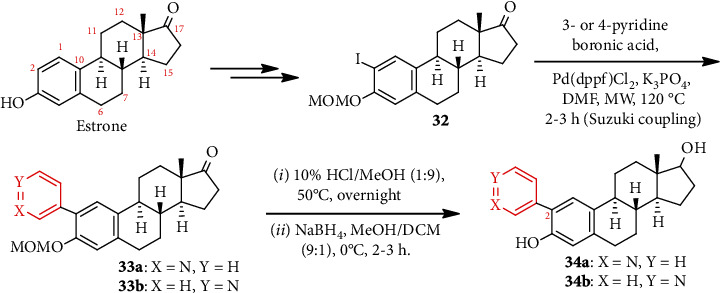
MW-assisted synthesis of estradiol substituted pyridine **34a, b**.

In an aim to develop cytochrome P450 (CYP) 1B1 inhibitors, two pyridinyl estradiols **34a, b** were synthesized (Scheme 7) [40]. Pyridine-3- and 4-boronic acid on MW irradiation with estronyl iodide **32** in the presence of K_3_PO_4_ and Pd(dppf)Cl_2_ (catalyst) underwent Suzuki coupling and formed **33a** and **33b**, respectively. Removal of MOM protecting group followed by reduction of C-17 carbonyl provided **34a, b**. Compound **34a** was found to be the most potent enzyme inhibitor (IC_50_ = 0.011 *μ*M).

#### 3.1.3. Green Synthesis

**Scheme 8:**
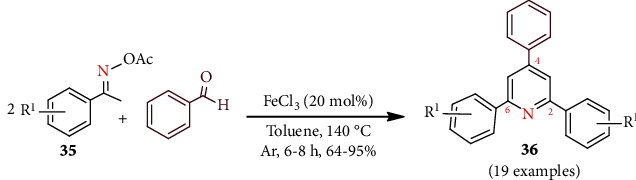
Fe-catalyzed green synthesis of pyridines from ketoxime acetates.

A facile green protocol for the preparation of substituted pyridines has been reported [41]. In this protocol, a novel, facile, and green conversion of ketoxime acetates **35** and benzaldehyde was conducted using FeCl_3_ as a catalyst, and chemoselective 2,4,6-trisubstituted symmetrical pyridines **36** were constructed (Scheme 8). Encouragingly, the reaction was completed greenly without any additives. Possible mechanisms of Fe-catalyzed cyclization of ketoxime acetates and aldehydes are also discussed.

#### 3.1.4. Miscellaneous Techniques

**Scheme 9:**
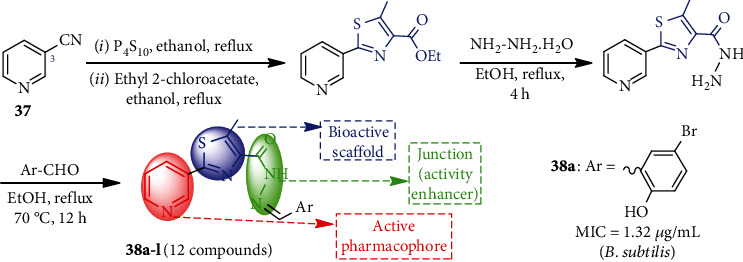
Synthesis of pyridine-3-thiazole hydrazides **38a-l**.

Kamat et al. [19] synthesized a new class of pyridine-3-thiazole hydrazides **38a-l** bearing thiazole and CONH moieties at the C-3 position starting from 3-cyanopyridine (**37**, Scheme 9). Most of the hydrazides have antibacterial efficacy against four tested bacterial pathogens.

**Scheme 10:**
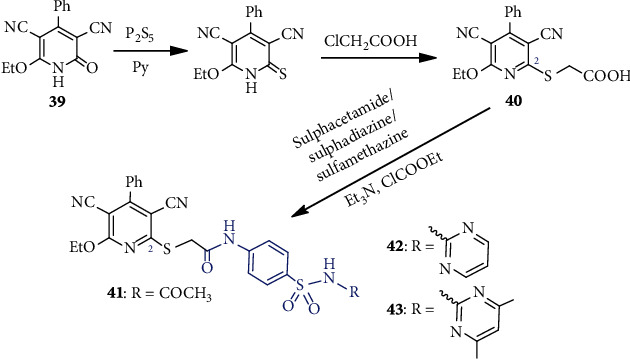
Synthesis of pyridine-based sulfa-drugs **41**-**43**.

El-Sayed et al. [42] synthesized three pyridine-based sulfa-drugs from *o*-hydroxy cyanopyridine derivative **39** (Scheme 10) for antimicrobial tests. Sulfurization of **39** followed by alkylation gave **40**, which on separate treatment with sulfacetamide, sulfadiazine, and sulfadimidine (sulfamethazine), furnished corresponding sulfonamides **41**, **42**, and **43**, respectively. Compounds **41-43** exhibited significant broad antimicrobial activities against four bacterial and two fungal pathogens.

**Scheme 11:**
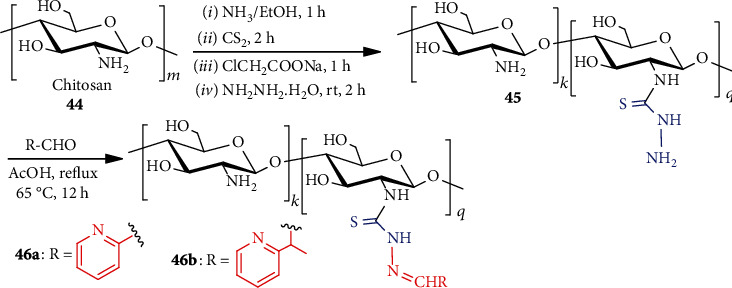
Synthesis of chitosan pyridine-thiosemicarbazones **46a, b**.

Considering biological interest, a simple strategy for the preparation of pyridine-based chitosan thiosemicarbazide **45a, b** was described [43]. Treatment of chitosan **44** with ammonia followed by carbon disulfide produced ammonium dithiocarbamate chitosan (ADC, **45**). This ADC, upon stirring with sodium chloroacetate, formed sodium carbethoxy dithiocarbamate chitosan, which was refluxed with pyridine-based carboxaldehyde and produced chitosan pyridine-2-thiosemicarbazones **46a** and chitosan 2-acetyl pyridine-2-thiosemicarbazones **46b** (Scheme 11). For efficient preparation, the overall process was conducted in one pot without the isolation of the intermediates. Compound **46a, b** was found biologically potential after further conversion and tests.

**Scheme 12:**
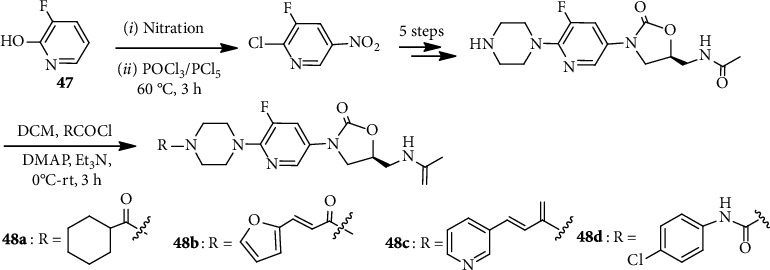
Linear synthesis of 3-(pyridine-3-yl)-2-oxazolidinone derivatives **48**.

Recently, a lengthy linear method was reported for the synthesis of a number of 3-(pyridine-3-yl)-2-oxazolidinone derivatives (Scheme 12) [44]. Starting from the readily accessible 3-fluoro-2-hydroxypyridine (**47**), the target oxazolidinone products (**48**; six examples) were prepared in eight steps. The antibacterial efficacy of these compounds **48a-d** was found to be comparable to the first oxazolidinone antibacterial agent, linezolid, and exerted strong inhibition against Gram-positive bacteria. In fact, **48d** showed a stable and longer resistance (15 days) on *S. pneumoniae* (ATCC 49619), considerably longer than that of linezolid antibiotic.

**Figure 3:**
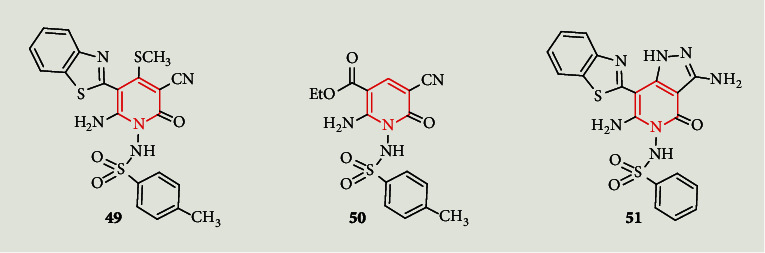
Structures of *N*-sulfonyl aminopyridines compound **49-51**.

In addition, a novel series of *N*-sulfonyl aminopyridines containing either a benzothiazole or benzimidazole ring was developed by Azzam et al. [45]. Of the twelve synthesized novel compounds, compounds **49** and **51** (Figure 3) showed excellent antimicrobial potential. The antimicrobial testing of the novel compounds also revealed that **49** and **50** displayed a greater inhibition zone against *Klebsiella pneumonia* than sulfadiazine and gentamicin. Moreover, compound **51** showed a higher inhibition zone compared to ampicillin against *Staphylococcus aureus*.

### 3.2. Ring-Fused Pyridine Analogs

#### 3.2.1. Catalyst-Mediated Synthesis

**Scheme 13:**
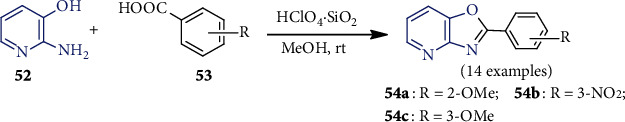
HClO_4_·SiO_2_ catalyzed synthesis of 2-(phenyl)oxazolo[4,5-*b*]pyridine **54**.

Silica-supported perchloric acid (HClO_4_•SiO_2_) catalyzed one-pot condensation between 2-amino-3-hydroxy pyridine (**52**) and substituted benzoic acids **53** furnished 2-(phenyl)oxazolo[4,5-*b*]pyridine derivatives **54** (Scheme 13). *In vitro* antibacterial tests established compounds **54a**, **54b**, and **54**c as strong inhibitors for methicillin-resistant *S. aureus* (MRSA, MIC: 1.56–3.125 *μ*g/mL) [46].

**Scheme 14:**
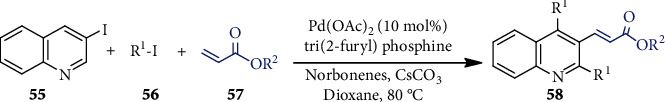
Pd-catalyzed synthesis of substituted quinolines.

The incorporation of functional moieties in the pyridine nucleus is essential to enhance its biological properties. In this regard, Fu et al. [47] reported a palladium and tri(2-furyl) phosphine-catalyzed alkylation of iodo-substituted quinoline with moderate to good yields (Scheme 14). The reaction proceeds with the Catellani reaction among iodoquinoline **55**, iodoalkane **56**, and *α*,*β*-unsaturated ester **57** and produced 2,3,4-trisubstituted-quinolines **58**.

**Scheme 15:**
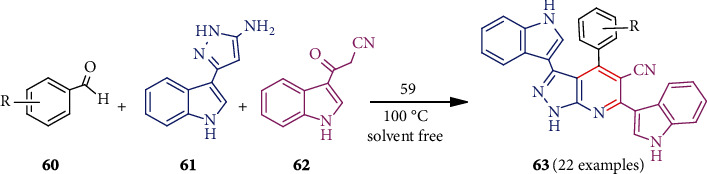
Synthesis of pyrazolopyridines applying Fe_3_O_4_@MIL-101(Cr)-N(CH_2_PO_3_)_2_ catalyst.

Fe_3_O_4_-derived novel catalyst namely Fe_3_O_4_@MIL-101(Cr)-N(CH_2_PO_3_)_2_ (**59**) was successfully applied as a catalyst for the preparation of medicinally significant novel pyrazolo[3,4-*b*]pyridines **63** [48]. Three-component condensation among aldehydes **60**, 5-(1*H*-indol-3-yl)-2*H*-pyrazol-3-ylamine **61**, and 3-(cyanoacetyl)indole **62** in the presence of this catalyst **59** without solvent at 100°C gave pyrazolo[3,4-*b*]pyridines in high yields (Scheme 15). The mechanism of such condensation by this novel catalyst was also proposed. In addition, such catalyst-promoted synthesis of **63** had advantages like a short reaction time, a clean profile of the reaction, and catalyst recyclability.

#### 3.2.2. Diels-Alder Reaction Strategy

**Scheme 16:**
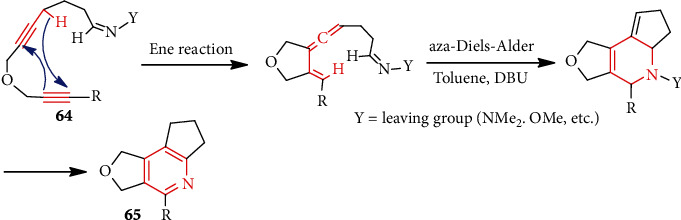
Synthesis of pyridines via aza-Diels-Alder strategy.

To overcome the challenge of rapid synthesis of multisubstituted or functionalized pyridines, the Diels-Alder reaction with *N*-containing dienophiles (azadienophiles) is described. In such a strategy, initially prepared vinylallene (*s*-*cis* conformation) via ene reaction can easily participate [4 +2] cycloadditions with azadienophiles (cyano groups, dimethylhydrazones, oximino ethers, etc.). For example, Hamzik et al. [49] succeeded in the synthesis of polycyclic pyridine **65** from **64** (Scheme 16).

**Scheme 17:**
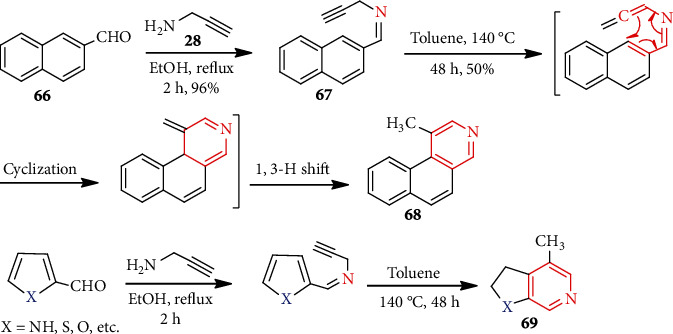
Synthesis of fused pyridine compounds.

Later, Şendil et al. [50] employed a strategy that was nearly identical to the above one to access pyridine-fused aromatic molecules. The 1-(naphthalen-2-yl)-*N*-(prop-2-yn-1-yl)methanimine (**67**) obtained from **66** underwent electrocyclization of the azatriene system and furnished polycyclic pyridine **68** (Scheme 17). Applying the same developed cyclization method, they prepared more heterocycle-fused pyridines (e.g., **69**).

**Scheme 18:**
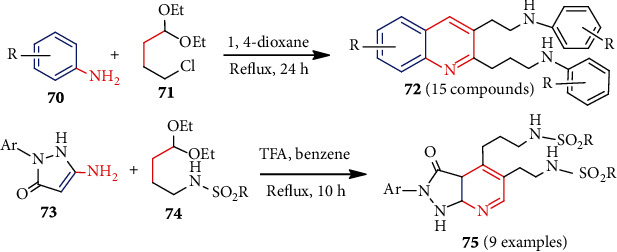
Synthesis of benzo[*b*]pyridines **72** and pyrazolo[3,4-*b*]pyridines **75**.

Very recently, Rizbayeva et al. [51] used aza-Diels-Alder reaction to synthesize functionalized new benzo[*b*]pyridine (quinoline) **72** and pyrazolo-pyridine **75** in a single step (Scheme 18). Reflux of a mixture of anilines **70** and 4-chloro-1,1-diethoxybutane **71** (3 : 2) in dioxane furnished 2,3-disubstituted quinoline **72** (~75%). Similarly, the interaction of aminopyrazolone **73** with *N*-(4,4-diethoxybutyl)sulfonamides **74** formed pyrazolo[3,4-*b*]pyridines **75**.

#### 3.2.3. Green Protocol

**Scheme 19:**
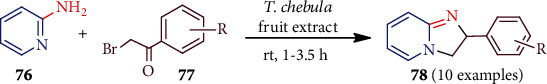
Green synthesis of 2-arylimidazo[1,2-*a*]pyridine catalyzed by plant extracts.

Green synthesis of 2-arylimidazo[1,2-*a*]pyridine **78** assisted by plant extracts is developed [52]. The condensation of 2-aminopyridine (**76**) with substituted phenacyl bromide (**77**) in the presence of *Terminalia chebula* fruit extract furnished **78** in high yield in a short time (Scheme 19).

**Scheme 20:**
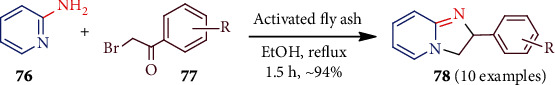
Green synthesis of imidazo[1,2-*a*]pyridines in presence of activated fly ash.

In the same year, Khansole [53] conducted the green synthesis of **78** using activated fly ash. The condensation of 2-aminopyridine (**76**) with substituted phenacyl bromides (**77**) in the presence of reusable activated fly ash afforded pyridines (**78**) (Scheme 20).

**Scheme 21:**
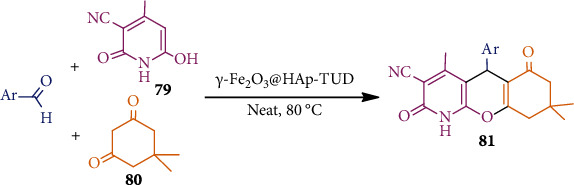
*γ*-Fe_2_O_3_@HAp-TUD mediated synthesis of chromeno[2,3-*b*]pyridines **81**.

Recently, recyclable *γ*-Fe_2_O_3_-based magnetite nanoparticles (MNPs) were synthesized from hydroxyapatite (HAp) and hexamethylene-1,6-diisocyanate followed by thiourea dioxide (TUD) (*γ*-Fe_2_O_3_@HAp-TUD) [54]. This *γ*-Fe_2_O_3_@HAp-TUD catalyst was used as a green catalyst for the preparation of chromeno[2,3-*b*]pyridines **81** (Scheme 21). Under solvent-free conditions, the reaction proceeds via one-pot, three-component reactions among 3-cyano-6-hydroxy-4-methyl-pyridin-2(1*H*)-one (**79**), aldehydes (Ar-CHO), and dimethoxydione (**80**). Compound **81** and its derivatives showed excellent *in vitro* antimicrobial activities, indicating their biomedical potential.

#### 3.2.4. Microwave-Promoted Synthesis

**Scheme 22:**
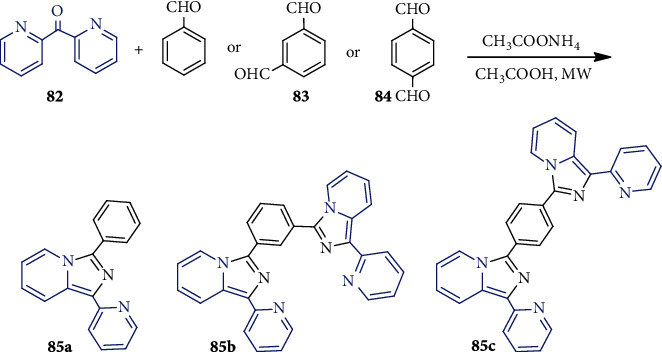
Synthesis of **85** using microwave-assisted cycloaddition.

Volpi et al. in 2016 [55] synthesized a series of pyridylimidazo[1,5-*a*]pyridine derivatives **85a-c** (Scheme 22). One-pot microwave-assisted condensation of substituted methanones **82** with benzaldehyde, isophthalaldehyde, or terephthalaldehyde **83** or **84** in the presence of NH_4_OAc produced **85a-c**. The method yielded water as a byproduct and occurred in the absence of any highly sensitive Lewis acids [56]. The imidazo[1,5-*a*]pyridine scaffolds containing compounds are found to have good antibacterial activities [57].

#### 3.2.5. Miscellaneous Method

**Scheme 23:**
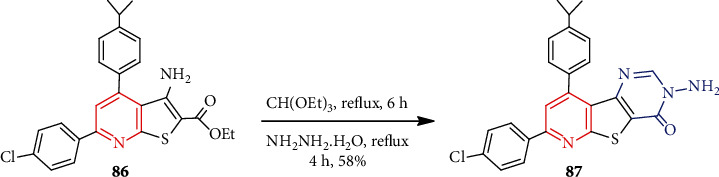
Synthesis of substituted thieno[3,2-*d*]pyrimidin-4(3*H*)-one **87**.

Several annulated thieno[2,3-*b*]pyridines synthesis was performed for antimicrobial interest [58]. Previously prepared thienopyridine **86** upon treatment with several reagents (PhNCS, HCONH_2_, NH_2_NH_2_, etc.) easily furnished corresponding thieno[3,2-*d*]pyrimidin-4(3*H*)-ones via nucleophilic addition. For example, **86** on treatment with triethyl orthoformate followed by hydrazine hydrate afforded compound **87** (Scheme 23). Compound **87** showed the highest zone of inhibition (29 mm) against Gram-positive *Staphylococcus aureus* and was comparable to cefotaxime (31 mm).

## 4. Antibacterial Properties of Pyridine-Based Compounds

As antibiotic resistance becomes an increasingly serious threat to public health, scientists are always on the lookout for new bacterial inhibitors. Most of the antibiotics now in use are becoming ineffective against bacterial infections [59, 60]. Therefore, the development of new, more potent antibacterial drug candidates is an urgent medical priority.

The incorporation of a pyridine motif into a pharmaceutical product can raise that product's biochemical potency and metabolic stability [61], as well as its permeability and difficulty in forming protein-binding interactions [62, 63]. There is a wide variety of medications in hospital use (Figure 1), and several drugs have been approved by the FDA since 2015 for cancer/HIV therapy, such as fostemsavir (2020), ivosidenib (2019), lorlatinib (2018), apalutamide (2018), and abemaciclib (2015). Encouragingly, the FDA has also approved several antibiotics stemming from the pyridine motif, including delafloxacin (Baxdela™; 2017), ceftazidime (Fortaz™; 2015), tedizolid (Sivextro™; 2014), and ceftaroline fosamil (Teflaro™; 2010) [5]. Encouraged by these results, many synthetic pyridines have been tested for their biological functionality since 2015. Some significant results are discussed herein.

In 2016, newly prepared several pyridine-imidazo[2,1*b*]-1,3,4-thiadiazole compounds were tested against seven microbial pathogens and showed good antimicrobial activity (maximum 95.1% inhibition) [64]. Later on, another new type of pyridine compounds, namely, 3-chloro-1-(4-substituted phenyl)-4-(pyridin-3-yl)azetidin-2-one compounds, were prepared for antimicrobial interest [65] and found that such compounds are potent against *S. aureus* (MTCC-3160). Polysubstituted 2-amino-4-aryl-3,5-dicarbonitrile-6-thiopyridines **88a-k**, synthesized by Koszelewski et al. [66], are found to inhibit *Escherichia coli* model strains K12 and R2–R4. MIC and MBC tests clearly demonstrated very low values of 0.2–1.3 *μ*g/mL and 4-45 *μ*g/mL, respectively. Compounds **88a, g, i, j** with different electron-withdrawing groups (EWG: CN, Cl, Br, and NO_2_) attached to the aromatic ring (at C-2 and C-4) enhance the *E. coli* inhibition process. They have shown that these compounds interact with bacterial cell walls irreversibly, leading to apoptosis. As a result, known antibiotics can be replaced with 2-amino-4-aryl-3,5-dicarbonitrile-6-thiopyridines.

**Figure 4:**
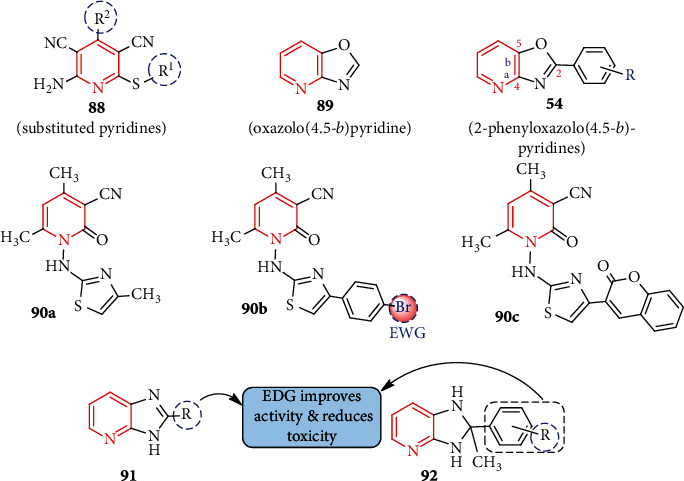
EWG and EDG effects of the substituents in different pyridine scaffold.

After synthesizing pyridine derivatives containing oxazolidinone, Jo et al. [67] found that they were very active against bacteria. Two antibiotic-resistant bacterial strains and a number of other Gram-negative and Gram-positive bacterial strains were tested for antibacterial activity with **54a-c ***in vitro* and *in vivo* [38] (Figure 4). Although the pyridine moiety may tolerate a wide variety of substituted (hetero)aromatic rings, the presence or orientation of methyl groups on the (hetero)aromatic rings significantly affected bacterial activity [68]. Another line of inquiry resulted in the development of antibacterial drugs by the manufacture of oxazolo[4,5-*b*]pyridine derivatives. A methicillin-resistant strain of *Staphylococcus aureus*, the causative agent of many hospital-acquired infections, was particularly susceptible to the activity of these chemicals [69]. Oxazolo[4,5-*b*]pyridine analogs were shown to be more efficient against Gram-positive bacteria than Gram-negative bacteria in subsequent studies. 2-Phenyloxazolo[4,5-*b*]pyridine was very efficient in killing methicillin-resistant *S. aureus*, with an activity of 1.56 to 3.12 *μ*g/mL. The MICs for older antibiotics like ampicillin and streptomycin, on the other hand, ranged from 6.25 to 12.5 *μ*g/mL. There is evidence that many different types of bacteria can be effectively combated with these chemicals [70, 71]. As an added bonus, *S. aureus* may secrete a type A staphylococcal enterotoxin protein. Additional *in vitro* and computational studies have shown that 2-phenyloxazolo[4,5-*b*]pyridine derivatives (**89**) are very active against bacteria, even when compared to conventional antibiotics like ampicillin and streptomycin (Figure 4). The compounds were then tested for ligand-protein binding affinity using the *S. aureus* (MRSA) protein, with results showing a higher affinity for binding than that of currently used drugs [72].

Dihydropyridine-containing thiazole derivatives were first examined using *in silico* molecular docking simulations for their potential DNA gyrase inhibitory action. Testing the substances in question for their capacity to kill germs was done to back up the study done on a computer [70]. The results showed that *N*-aminothiazolyl-1,2-dihydropyridine with a substituent at the 4-position of thiazolyl group **90a-c** (Figure 4) showed better antibacterial potentiality against the tested four bacteria (*B. subtilis*, *E. coli*, *P. aeruginosa*, and *S. aureus*) than the standard antibiotic ampicillin. The existence of the electron-withdrawing group (EWG) (as in **90b**) in the phenyl ring attached to the thiazole part could be responsible for better inhibition and activity.

Among the ring-fused pyridines, synthetic novel 3*H*-imidazo[4,5-*b*]pyridine (11 compounds) and 1*H*-imidazo[4,5-*b*]pyridine (10 compounds) analogs were subjected to *in vitro* antitubercular activity against *M. tuberculosis* (H37RV) [73]. Compounds **91a-c, j** (3.125 *μ*g/mL) and **92a, c, f** (3.125-6.25 *μ*g/mL) were found highly active and comparable to pyrazinamide (MIC = 3.125 *μ*g/mL) and streptomycin (MIC = 6.25 *μ*g/mL). The electron-donating group(s) (EDG) attached to the imidazole ring probably reduced cytotoxicity and enhanced antibacterial functionality. Thus, these imidazo-fused pyridines might develop bacterial-related multidrug-resistant (MDR) infections caused by *M. tuberculosis*.

## 5. Conclusion

The pyridine skeleton generates a suite of flexibility, leading to the formation of libraries of compounds bearing a variety of functional groups. This is due to its characteristic solubility, basicity, and ability to form hydrogen bond-formation chemistry, which led to the bioisostere of amides, amines, and *N*-containing heterocycles. All these characteristics make this pyridine skeleton a significant unit in a plethora of drugs and pharmaceuticals. Thus, many improved/novel methods have been reported/developed for the synthesis of functionalized pyridines since 2015. The available antibacterial results concluded that (*i*) polysubstituted and ring-fused pyridines exhibited considerable antibacterial properties, including methicillin-resistant *S. aureus* (MRSA) and (*ii*) EWG in substituted pyridines and EDG group in ring-fused pyridines were found to enhance antibacterial potentiality. In spite of the pyridine scaffold bearing overwhelming drug candidates, thoughtful research is essential to overcome drug resistance and side effects. Chemistry, synthesis, and antibacterial potential as highlighted in this minireview may promote better understanding and further effective research of the ever-expanding pyridine scaffold in medicinal chemistry.

## Data Availability

All data are available on request.
